# Radiotherapy in the treatment of aggressive fibromatosis: experience from a single institution

**DOI:** 10.1186/s13014-020-01565-9

**Published:** 2020-06-05

**Authors:** K. Seidensaal, S. B. Harrabi, F. Weykamp, K. Herfarth, T. Welzel, G. Mechtersheimer, B. Lehner, M. Schneider, S. Fröhling, G. Egerer, J. Debus, M. Uhl

**Affiliations:** 1 Department of Radiation Oncology, Heidelberg University Hospital, Im Neuenheimer Feld 400, 69120 Heidelberg, Germany; 2 Heidelberg Institute of Radiation Oncology (HIRO), Heidelberg, Germany; 3 National Center for Tumor diseases (NCT), Heidelberg, Germany; 4 Heidelberg Ion-Beam Therapy Center (HIT), Department of Radiation Oncology, Heidelberg University Hospital, Heidelberg, Germany; 5 Clinical Cooperation Unit Radiation Oncology, German Cancer Research Center (DKFZ), Heidelberg, Germany; 6 Institute of Pathology, University of Heidelberg, Heidelberg, Germany; 7 Center for Orthopedics, Trauma Surgery and Paraplegiology, University of Heidelberg, Heidelberg, Germany; 8 Department of General, Visceral and Transplantation Surgery, University Hospital Heidelberg, Heidelberg, Germany; 9 Department of Translational Medical Oncology, National Center for Tumor Diseases Heidelberg and German Cancer Research Center, Heidelberg, Germany; 10 Department of Hematology, Oncology and Rheumatology, Heidelberg University, Heidelberg, Germany; 11 German Cancer Consortium (DKTK), partner site Heidelberg, Heidelberg, Germany

**Keywords:** Aggressive fibromatosis, Desmoid tumor, Radiotherapy, Proton therapy

## Abstract

**Background:**

Desmoid-type fibromatosis is a rare, potentially locally aggressive disease. Herein we present our experience in the treatment with radiotherapy.

**Methods and materials:**

In total 40 patients who received 44 treatments from 2009 to 2018 at the Heidelberg University Hospital with photons (*N* = 28) as well as protons (*N* = 15) and carbon ions (*N* = 1) were investigated. The median age at radiotherapy was 41 years [range 8–78]. Familial adenomatous polyposis (FAP) was confirmed for nine patients and 30 had a unifocal desmoid tumor. The localizations were abdominal wall, abdominopelvic cavity, thoracic wall, extremity, head and neck and trunk. The median prescribed dose was 54 Gy/ Gy (RBE) [range 39.6–66, IQR 50–60]. Eleven treatments were performed at the time of first diagnosis; 33 at the time of progression or recurrence. Post-operative radiotherapy was performed in 17 cases. The median planning target volume was 967 ml [84–4364 ml, IQR 447–1988]. Survival analysis was performed by the Kaplan-Meier Method.

**Results:**

The median follow-up time was 32 months [1–153]. At the end of the follow-up interval all patients but one were alive. The estimated local progression free survival of the treated lesion in 3 and 5 years was 76.4% and 63,8%, respectively. The progression-free survival in 3 and 5 years was 72.3 and 58.4% and the overall survival was 97.4 and 97.4%, respectively. In case of macroscopic tumor (*N* = 31) before radiotherapy a partial remission was observed in 12 cases (38.7%) and a complete remission in 4 cases (12.9%). Progression was observed in 13 (29.5%) cases, predominantly at the margin of the planning target volume (PTV, *N* = 5, 38,4%) followed by progression within the PTV (*N* = 4, 30.8%). In univariate analysis multifocal localization was associated with impaired progression-free survival (*p* = 0.013). One patient developed a grade V gastrointestinal bleeding, otherwise no acute toxicity >°III was observed. Late toxicity was depending on the localization of the desmoid tumor and was especially severe in patients with FAP and abdominopelvine desmoids including gastrointesinal fistula, perforation and abscess.

**Conclusion:**

Radiotherapy in the treatment of desmoids can lead to long term control. Treatment of patients with abdominopelvine desmoids should be avoided, as the risk of higher-grade complications is substantial.

## Introduction

Aggressive fibromatosis describes rare benign lesions, which arise from musculoaponeurotic structures. The alternative term “desmoid” refers to the tendon like appearance. They account for 0.03% of new diagnosed neoplasms and for 3% of soft tissue tumors [1]. The treatment options include surgery, radiotherapy, hormonal therapy, chemotherapy and anti-inflammatory agents [2]. Despite their clinically circumscriptive appearance, histologically those tumors show diffuse and infiltrative growth into surrounding tissues and are not encapsulated [3]. The course of the disease is variable and unpredictable including growth, progression, stabilization, and even spontaneous regression [4]. Thus, for most patients an observation period is advisable [5]. When active therapy is necessary, surgery is one option. Wide microscopic margin-negative resection (R0) is the goal, but conservation of cosmesis and function is the major priority [6]. Despite the tendency for local recurrence, desmoid tumors do not metastasize but they can present multifocal in the same limb or region. Preceding traumatic events to the involved area, previous pregnancy as well as surgical scars have been identified as factors contributing to the development of aggressive fibromatosis. Desmoids occur sporadic but are also associated with familial neoplastic syndromes. Especially at the abdominal site desmoids are often encountered in patients with familial adenomatous polyposis coli (FAP, Gardners syndrom). Treatment of aggressive fibromatosis is complex and depending on the anatomical location and size of the tumor. Mortality might be low but the risk of significant disability and morbidity is crucial [7]. Herein we present our single center experience in the treatment of desmoid tumors by radiotherapy, the focus of the study is to assess i) treatment related morbidity, ii) patterns of response or treatment failure, and identify iii) opportunities to improve treatment strategies.

## Methods and materials

### Data collection

The Ethics committee of the University of Heidelberg approved the study. Data on patients’, tumor and treatment characteristics, imaging modalities/ results and outcome were obtained from retrospective review of medical records. The following variables with the according thresholds were analyzed for their prognostic value: gender, age at radiotherapy (median of 41y), FAP (yeas/no), tumor size described by PTV (median of 967 ml), tumor site (abdominal vs. extraabdominal), timing of treatment (primary situation vs. recurrence/progression), macroscopic tumor at the time of treatment (yes/no), applied dose (median of 54 Gy/Gy (RBE) and threshold of 50Gy/Gy (RBE)), previous surgery (yes/no), previous medical treatment (yeas/no), particle vs. photon radiotherapy and definitive radiotherapy vs. postoperative radiotherapy. .

### Study cohort

**Fig. 1:**
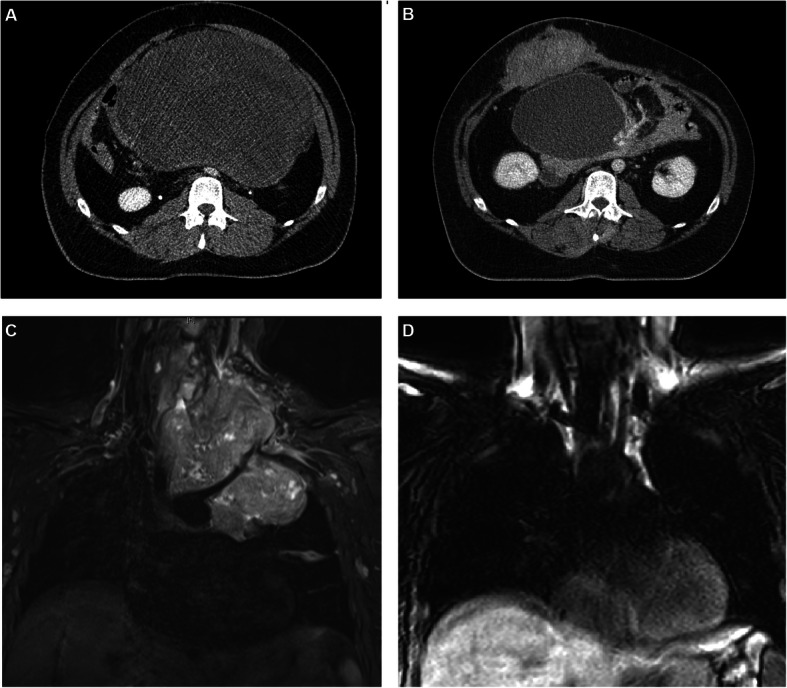
Examples of patients with desmoid tumors: A 27-year-old patient with a progressive, gigantic, FAP-associated mesenteric desmoid tumor, treatment was performed with photon-IMRT up to 50.4 Gy in 28 fractions, B: The patient developed a new symptomatic desmoid tumor of the abdominal wall 7 years after the primary treatment and was treated with protons 56 Gy (RBE) in 28 fractions. The mesenteric desmoid regressed in size, and stabilized with a cystic appearance on CT and MRI (not shown). C: Thirty-two-year-old patient who was pregnant at diagnosis of a gigantic cervico-thoracic desmoid tumor. The patient was treated beginning in the 28th week of pregnancy with protons, however the tumor progressed rapidly and the treatment was discontinued at 30 Gy (RBE). Caesarean section was performed and treatment with methotrexate and vinorelbine initiated. The tumor regress significantly after that

Forty consecutive patients who have been treated at the University Hospital Heidelberg August 2009–December 2018 were included in this study. One patient received a previous treatment at our intuition in April 2006 which was also considered. In total the patients received 44 courses of radiotherapy. Of the four patients treated twice, three patients developed new desmoids in proximity to the previous lesion (intraabdominal/ abdominal wall *N* = 2, lower extremity *N* = 1, example Fig. 1 A and B), and one patient progressed at the cranial margin of the previous desmoid tumor. Five patients were excluded as no follow up was available, two previous courses of radiotherapy dated back longer were not included as the data was insufficient for analysis. The median age at first presentation was 41 years [8–78], 30 patients (75%) had one single desmoid. The treated lesions were located in the abdominopelvic cavity (*N* = 11, 27.5%), thoracic wall (*N* = 9, 22.5%), extremities (*N* = 9, 22.5%), abdominal wall (*N* = 4, 10%), trunk (*N* = 4, 10%) and head and neck (*N* = 3, 7.5%). Nine patients had a familial adenomatous polyposis (FAP, Gardner’s syndrome), the diagnosis desmoid tumor of five of that was based on morphological imaging and not histologically confirmed. Five FAP patients had previous surgery in the region of the desmoid tumor, thereof four a prophylactic proctocolectomy and one a gastrectomy (Table 1).

**Table 1:** Patient characteristics

	n	Percent
Number of patients	40	
Number of treatments	44	
Gender		
Female	25	62.5
Male	15	37.5
Age at diagnosis		
Median (range)	39	1–78
Age at radiotherapy		
Median (range)	41	8–78
Multifokal/ unifokal at first RT		
Unifokal	30	75
Multifokal	10	25
Localization of treated tumor at first treatment		
Abdominopelvic cavity	11	27.5
Thoracic wall	9	22.5
Extremity	9	22.5
Abdominal wall	4	10
Trunk (paravertebral)	4	10
Head and neck	3	7.5
Familial adenomatous polyposis (FAP)		
No	31	77.5
Yes	9	22.5
Previous surgery in area of primary tumor		
No (all/ thereof FAP patients)	35/4	87.5/ 10
Yes (all/ thereof FAP patients)	5/5	12.5/ 12.5
Histologically confirmed desmoid tumor		
Yes (all/ thereof FAP patients)	35/ 4	87.5/ 10
No (all/ thereof FAP patients)	5/ 5	12.5/ 12.5
Pregnant at radiotherapy		
Yes	1	2.5

The majority of treatments was performed not at the time of first diagnosis (*N* = 11, 25%) but at the time of progression or recurrence (*N* = 33, 75%). Twenty-seven treatments were performed by radiotherapy only and 17 as postoperative additive radiotherapy for microscopic (R1) or macroscopic (R2) incomplete resection. In 31 cases gross tumor was detectable on planning computed tomography.

Previous treatment included surgery and medical treatment and are summarized in Table 2. Only 14 (31.8%) had no prior surgery, 18 (40.9%) had one prior resection of the desmoid tumor, six (13.6%) two prior resections and six (13.6%) more than two prior resections. Seven treatments were performed as a second course of percutaneous radiotherapy, five had a clear overlap of the high dose area, one had no overlap and one was in a different region. Two patients with thoracic desmoids had previous radiotherapy to the chest for breast cancer.

In total 25 patients received no previous medical treatment, the rest of the cohort received various substances and combinations, including sulindac (*N* = 9), meloxicame (*N* = 2), celecoxib (*N* = 1), diclophenac (*N* = 1), tamoxifen (*N* = 7), raloxifene (*N* = 1), toremifen (*N* = 1), imatinib (*N* = 5), methotrexathe (*N* = 2), vinchristine (*N* = 2), doxorubicin (N = 5), actinomycin D (*N* = 1), cyclophosphamide (*N* = 1), dacarbazine (*N* = 3), One patient received a multiagent-chemotherapy, details on the substances were not available.

**Table 2:** Radiotherapy timepoint and concept, previous treatment characteristics

	n	Percent
Number of treatments	44	
Treatment timepoint		
Recurrence/ progression	33	75
At first diagnosis	11	25
Treatment concept		
Definitive RT	27	61.4
Direct post-operative RT	17	38.6
-Microscopically incomplete resection (R1)	8	18.2
-Macroscopically incomplete resection (R2)	5	11.4
-Unknown (Rx)	4	9.1
Foregoing surgery of the treated desmoid tumor		
No	14	31.8
1 resection	18	40.9
2 resections	6	13.6
> 2 resections	6	13.6
Macroscopic tumor before RT on planning computed tomography		
Yes	31	70.5
No	13	29.5
Previous radiotherapy		
Percutaneous RT of desmoid tumor	7	16.9
Overlap of high dose area	5	11
Previous medical treatment		
No	26	59.1
Yes	18	40.9

### Treatment planning and radiotherapy details

Patients were treated at Heidelberg Ion-Beam Therapy Center (HIT) and the Heidelberg University Hospital. Treatment characteristics are summarized in Table 3. Treatment with protons and carbon ions was performed exclusively with active raster scanning as previously published [8, 9]. Treatment with photons was performed as 3D conformal radiotherapy or intensity modulated radiotherapy (IMRT), either helical IMRT or IMRT in VMAT technique at the discretion of the according radiation oncologist. Intraoperative electron radiation therapy (IOERT) was performed by The Mobetron® (IntraOP Medical), a self-shielding, mobile linear accelerator. Gross Tumor Volume (GTV) included the gross tumor based on CT and MRI imaging, if available. The Clinical Target Volume (CTV) was defined as GTV plus surrounding areas at risk for containing microscopic disease. The CTV included the GTV aiming at a margin of 1–4 cm if feasible, depending on the location and anatomy. The CTV margins were smaller if the GTV was adjacent to the critical normal organs like small bowel. For extremity desmoids one third of the skin circumference was spared in order to reduce the risk of chronic lymph edema. The PTV margins depend on localization and technique, for cranial localization a 3 mm margin was added to the CTV, extracranial 5 mm in most cases, for particles 7 mm in beam direction and 5 mm in the other directions. Proton therapy was preferred when technically possible and covered by patient’s insurance (*N* = 15, 34%). Carbon ion radiotherapy was used in one case of re-irradiation. Photon radiotherapy was performed in the remaining cases (*N* = 28, 66%). The median prescribed dose of the percutaneous radiotherapy was 54 Gy/ Gy (RBE) (range 39.6–66, IQR 50–60). The median applied dose was 54 Gy/ Gy (RBE) (range 30–66, IQR 50–60). Four patients had a preliminary termination of treatment, three for reasons other than radiotherapy. Four patients hat an IOERT boost with the current treatment (12 Gy). The median planning target volume was 967 ml (range 84–4364 ml, IQR 447–1988).

**Table 3:** Treatment characteristics

		N = 44	Percent
RT Technique			
Photon		28	66
3-D conformal RT		8	18.2
IMRT		20	45.5
Particle		16	36.4
Protons		15	34
Carbon ion		1	2.3
Prescribed total dose (Gy/ Gy (RBE))			
Median (Range, IQR)		54	39.6–66, 50–60
< 50		5	11
≥ 50		24	55
≥ 60		15	34
Single dose			
Proton	1.8 Gy/ Gy (RBE)	14	31.8
Photon	2 Gy/ Gy (RBE)	29	65.9
Carbon ion	3 Gy (RBE)	1	2.3
Volume reduction (percutaneous boost)		4	9.1
Intraoperative electron radiation therapy (IOERT) boost			
	12 Gy	4	8.4
Planning target volume (ml)			
	Median (range, IQR)	967.06	(84–4364, 447–1988)
	Missing	1	2.3
Premature discontinuation of RT		4	9.1

### Statistical analysis

Follow-up time was calculated from the beginning of treatment until the last presentation. Local progression free survival (LPFS) was defined as the time from beginning of treatment until progression of the target lesion. PFS was defined as the time from beginning of treatment until progression of the target lesion, other lesions, new lesions or death. Survival analysis war performed from beginning of treatment until the first progression (*n* = 40), the second treatment was not considered. Survival curves were calculated by the Kaplan-Meier method and compared by the log-rank test. Variables with a *p*-value of less than 0.05 were considered significant. Response of the target lesion was defined according to the Response Evaluation Criteria in Solid Tumors (RECIST 1.1) and evaluated at the end of the follow-up interval on the last available MRI or CT. Toxicity was assessed by Common Terminology Criteria for Adverse Events (CTCAE) v. 5.0, transferred to this version when previously graded with former versions, when grading was not available, the toxicity was stated as present.

## Results

### Survival analysis

**Fig. 2:**
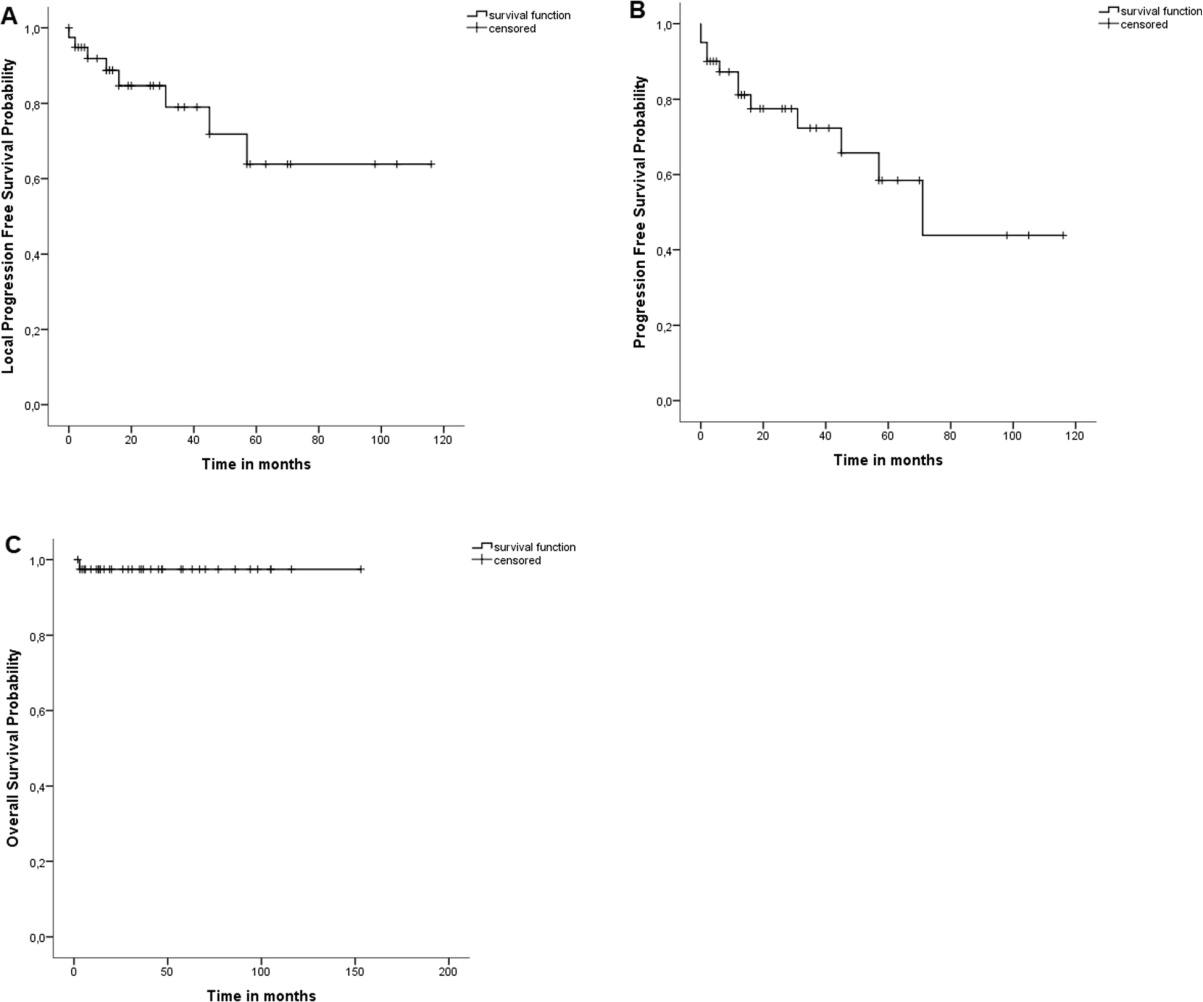
Survival analysis of patients with aggressive fibromatosis treated by radiotherapy: A) Local progression free survival, B) Progression Free Survival and C) Overall Survival

The median follow-up time was 32 months [range 1–153 months]. The local PFS was 79,4 and 63.8% at 3 and 5 years, respectively. The PFS from desmoid tumor of any location was 72.3 and 58.4% at 3 and 5 years, respectively. All but one patient were alive during the follow-up interval, resulting in an overall survival probability of 97.4% at 3 and 5 years (Fig. 2). In univariate analysis unifocal localization of the desmoid tumor was associated with superior PFS and age below the median of 41y with inferior PFS (*p* = 0.013 and *p* = 0.002, respectively, Supplemented Table 3). PFS did not differ significantly between patients with and without FAP, between patients with definitive vs. directs postoperative treatment as well as between the treatment at the time of progression or the time of the first diagnosis.

### Response analysis

Follow-up imaging was available for 43 of 44 treatments, as one patient died soon after the end of radiotherapy. Of 31 patients with macroscopic tumor at beginning of treatment 12 showed a partial remission of the tumor size (38.7%) and four a complete remission (12.9%). Stable disease was observed in eight cases (25.8%) of this subcohort and progressive disease in six (19.4%). A lower portion of treatments (*N* = 13, 29.5%) was performed as postoperative radiotherapy after incomplete resection and had no/ unsure (=differentiation between possible residual tumor and postoperative surgical changes was not exactly feasible) macroscopic tumor on planning computed tomography, those patients showed complete remission in 10 (77%) cases and progressive disease in three (23%) cases (Table 4).

**Table 4:** Outcome characteristics

		n = 44	Percent
Response of target lesion **with macroscopic tumor** before RT		31	70.5
	Complete Remission	4	12.9
	Partial remission	12	38.7
	Stable disease	8	25.8
	Progressive disease	6	19.4
	Missing	1	3.2
Response of target lesion **without macroscopic tumor** before RT		13	29.5
	Complete remission	10	77
	Progressive disease	3	23
Patterns of recurrence		13	29.5
	At the PTV Margin	5	38.4
	Within the PTV	4	30.8
	Same anatomical region, outside the PTV	2	15.4
	Different anatomical region	2	15.4

Recurrence occurred in 13 treatments. The pattern of recurrence was recurrence at the PTV margin (*N* = 5, 38.4%), recurrence within the PTV (*N* = 4, 30.8%) and outside the PTV (*N* = 2, 15.4%) or in different anatomical region (*N* = 2, 15.4%, Table 4).

### Toxicity

Late toxicity depended on the localization, patients with extraabdominal desmoids mostly suffered from lymphedema (*N* = 8), fibrosis (*N* = 4), decreased range of joint motion (*N* = 7) or sensory disturbance/paresis (*N* = 4) (Supplemented Table 2).

Patients with FAP and intraabominal desmoid tumors had a tendency for severe complications. A 44-year-old patient developed chylous ascites after radiotherapy for intraabdominal multilocalized desmoid tumors. He developed peritonitis and required opening of an abscess after paracentesis. Several times the patient developed sepsis which was contributed to cholangitis. In the location of the previous desmoid he developed an interenteric retention due to fistula. After five consecutive operations for persisting multiple fistula and perforations a jejunostomy was performed and the patient received a total parenteral nutrition (Supplemented Table 1).

One female patient (age 33 at the time of treatment) developed an abscess and a chronic inflammation in the location of the treated desmoid tumor which formed a fistula and a persisting ulcus to the neighboring ileo-anal pouch.

The treatment of one FAP patient with a central superinfected desmoid tumor was discontinued at 34.2 Gy before reaching the prescribed dose due to further progression of the abscess and progression in size of the desmoid tumor.

One patient with FAP, who has been treated for abdominopelvic aggressive fibromatosis, developed rectal bleeding and required cardiopulmonary resuscitation on the last day of radiotherapy. Furthermore, he developed a fistula between the treated desmoid and the adjacent bowel. He received drainage, however sepsis persisted. He died 2 months after the end of radiotherapy in palliative care.

Besides that, there were three further discontinuations, two for reasons other than the desmoid or its treatment and one was the case of a female patient who was pregnant at diagnosis of a gigantic cervico-thoracic desmoid tumor which presented with immense neuropathic pain due to damage of the cervical plexus, stricture of the upper respiratory tract and difficulties in breathing and swallowing. The patient was treated beginning in the 28th week of pregnancy with protons, however the tumor progressed rapidly. Consequently, the treatment was discontinued and Caesarean section was performed. Under the treatment with methotrexate and vinorelbine the tumor regressed significantly after birth (Fig. 1 C and D).

## Discussion

Desmoid tumors are rare and large or randomized series are not available up to date, thus there is no evidence-based approach. Characteristically the course of the disease is variable and difficult to predict.

In recent years immediate surgery especially for asymptomatic or paucisymptomatic desmoid tumors has been increasingly restricted 7 [2], as it has been observed that watchful waiting can achieve PFS rates of 50% in 5 years. A conservative, nonaggressive policy has been based on the ability of extra abdominal desmoid tumors to spontaneously stabilize [10–12]. Even spontaneous regressions are possible in 20% of cases, thus the tendency to follow with observation only is growing [5].

When local therapy is necessary surgery or radiotherapy or both form the options. Preservation of function is a priority; mutilating surgery has to be avoided and radiotherapy can be beneficial in selected cases esp. in the treatment of head and neck or intrathoracic desmoids [2]. A comparative review of 22 articles published by Nuyttens et al. showed that local control after treatment with a combination of surgery and radiotherapy was significantly improved compared to surgery alone (75% vs 61%), this applied to surgically positive and negative margins as well as primary and recurrent tumors. This study (like most) excluded articles with FAP, children, abdominal localization and head and neck because here response to treatment can be less or more favorable [13]. In our cohort younger age and multilocal localization was associated with worse survival, FAP showed however no significant difference. A second more recent meta-analysis of in total 1295 patients showed that the risk of local recurrence was twofold higher for those with positive resection margins. Adjuvant RT was only beneficial in case of incomplete resection for primary but particularly for recurrent tumors [14]. On the other hand, it has been shown for desmoid tumors of the abdominal wall that of 41 patients only one developed a recurrence within 97 months after incomplete (R1) resection [4]. To date the benefit for postoperative radiotherapy is not conclusively clarified. In cases of recurrence after previous surgery and when local control is crucial, postoperative radiotherapy can be considered. It is worth noting that a significant portion our cohort (*N* = 17) was presented after incomplete resection and 12 patients were presented with recurrent disease after two or more resections. Previously no significant differences in terms of local control were reported for radiotherapy alone compared to the combination of radiotherapy and surgery [15]. Local control after radiation therapy was shown to be better for patients with fewer than two operations versus more than three [16]. Additionally, recurrence seems to be a significant unfavorable independent risk factor for further recurrence [17]. Taking all this into account, one might consider definitive radiotherapy when the risk of incomplete resection is high in order to avoid the need of two subsequent treatment modalities and the combination of the according toxicities.

A phase II pilot study (*n* = 44) for patients of inoperable progressive desmoid tumors of the trunk and extremities established a dose of 56 Gy in 28 fractions. The response rates of the cohort were similar to our subcohort of patients with macroscopic tumors at beginning of radiotherapy regarding partial remission (36.4%) and complete remission (13.6%) [18]. However, the rate of stable disease was higher compared (25.8% vs 40.9%) und the rate of progressive disease lower (19.4% vs. 6.8%). Compared to many tumors of mesenchymal origin this reduction in size is striking, as in many tumors with low proliferation indices (e.g. low-grade chondrosarcoma, liposarcoma) a stationary size with assumed biological inactivation characterize the treatment response of the tumor. Similar local control rates of 79% after a median follow-up of 44 months were reported by the German Cooperative Group on Radiotherapy for Benign Diseases (GCG-BD) [19].

Proton therapy has a superior dose distribution compared to percutaneous photon therapy and allows a better sparing of surrounding organs at risk with potential in reducing acute and long-term toxicity. Retrospective data on a cohort of 115 desmoid patients treated between 1965 and 2005 showed a rate of radiation related complications of 17% with a median follow-up of 10.1 years [15], the potential of particle therapy to reduce this rates has to be investigated further in future. The main concern about radiotherapy is the risk of secondary malignancy e.g. radiation induced sarcoma in a young population with substantial chances of long term survival. The medium and low dose of protons to surrounding tissue is significantly lower on dosimetric comparison and thus the risk of secondary malignancy is reduced [20]. In clinical cohorts of adults as well as pediatric patients low rates of secondary malignancies have been reported for the treatment with protons [21, 22]. In a large case matched comparison, the rates were 5.2 and 7.5% for the proton and photon cohort, respectively [21]. Whenever available and technically possible we thus strongly recommend the use of proton therapy, in analogy to the recommendations for pediatric patients. In case of re-irradiation we tend to rely on carbon-ions as the lateral dose gradients are even stepper than those of protons and thus treatment related morbidity is reduced [23]. Aside from the case of re-irradiation no recommendations can be made regarding carbon ion therapy due to the very limited experience.

Historically we aimed at prescription doses of approximately 60 Gy in parallel to other tumors of mesenchymal origin. However, close location to sensitive organs at risk made a reduction of the dose necessary and we achieved in this cohort a median dose of 54 Gy/ Gy (RBE) with the here presented promising results. The latest consensus guidelines of the Desmoid Working Group suggest a “moderate dose radiotherapy” in those cases when active therapy is necessary but the risk of surgery-associated long-term morbidity is high [6]. In parallel, the previous European Consensus Guideline recommended 56 Gy in 28 fractions, based on the result of the aforementioned phase II study [2]. It is worth noting that a dose of 50 Gy/ Gy (RBE) or lower was associated with impaired survival on univariate analysis in our cohort (*p* = 0.014), thus our data support a prescription dose of ~ 56 Gy/ Gy (RBE).

The risk of progression during pregnancy is high, however a desmoid tumor is not a contradiction for future pregnancies. Although it is described that less than 50% of patients require treatment [24], this was not the case with the patient described here due to the gigantic size and rapid progression of the tumor compressing the upper airways and esophagus. We included this seldom case in our analysis, to give an impression on the level of local aggressiveness a desmoid tumor can develop.

FAP- associated desmoid type fibromatosis has to be differed from the sporadic tumors. Commonly they are larger, multiple and often intra-abdominal. The risk of recurrence is furthermore higher (44% vs. 25%) and among the FAP-patients desmoids constitute a significant cause of death [25]. The treatment is even more challenging. Tumor related complications are common and include intestinal obstruction, perforation or ischemia as well as urethral obstruction [26]. We observed severe acute and late toxicity with radiotherapy for FAP-associated desmoid tumors in our series. Necrosis, abscess formation [27, 28] and fistula has been described previously for desmoid patients that have not been treated by radiotherapy. Our data show that radiotherapy although highly effective in the reduction of size cannot prevent and might even promote the formation of these complications. Based on this experience we conclude that RT in patients with FAP-associated desmoid tumors should be only initiated when other options have been exploited.

## Conclusion

The limitations of the study lie in the retrospective character. We deliberately did not exclude patients with head and neck desmoids, FAP-associated desmoids or juvenile desmoids, which can influence the estimated local progression free survival [25, 29, 30], however provides more comprehensive information regarding treatment related morbidity. Aside of FAP-associated desmoids, RT was safe and feasible and contributed to local control with good chances of even reduction in tumor size. Limited evidence hinders the establishment of unequivocal guidelines; thus, treatment decisions have to be obtained interdisciplinary and a personalized management considering the varying locations and the possibly associated tumor-related and treatment-related complications has to be established.

## Supplementary information

**Additional file 1.**

## Data Availability

The datasets used and/or analyzed during the current study are available from the corresponding author on reasonable request.

## Abbreviations

CTCAE: Common Terminology Criteria for Adverse Events
CTV: Clinical target volume
GTV: Gross tumor volume
FAP: Familial adenomatous polyposis
GCG-BD: German Cooperative Group on Radiotherapy for Benign Diseases
HIT: Heidelberg Ion-Beam Therapy Center
IMRT: Intensity modulated radiotherapy
LPFS: Local progression free survival
PFS: Progression free survival
PTV: planning target volume
OS: Overall survival
RBE: Relative biological effectiveness
RT: radiotherapy
VMAT: Volumetric modulated arc therapy
