# Supplementary material for: Radiotherapy in the treatment of aggressive fibromatosis: experience from a single institution
Source: Radiat Oncol. 2020 Jun 5;15:143. doi: 10.1186/s13014-020-01565-9 (PMC7275436; doi:10.1186/s13014-020-01565-9)
Supplement: Supplementary file 1 — Additional file 1. [file 13014_2020_1565_MOESM1_ESM.docx]

| **Supplemented Table 1:**  **Acute toxicity (<=3 months)** | | | | | | | |
| --- | --- | --- | --- | --- | --- | --- | --- |
| Toxicity | All | Present | CTCAE Grade | | | | |
|  |  |  | I | II | III | IV | V |
| Radiodermatitis | 29 |  | 22 | 3 | 4 |  |  |
| Hyperpigmentation | 9 | 5 | 4 |  |  |  |  |
| Lymphedema | 5 | 2 | 2 | 1 |  |  |  |
| Pruritus | 2 | 2 |  |  |  |  |  |
| Dysphagia | 1 | 1 |  |  |  |  |  |
| Diarrhea | 5 | 2 | 1 | 1 | 1 |  |  |
| Meteorism | 1 | 1 |  |  |  |  |  |
| Nausea | 3 | 1 |  | 2 |  |  |  |
| Vomiting | 1 | 1 |  |  |  |  |  |
| Loss of appetite | 1 | 1 |  |  |  |  |  |
| Vaginal mucositis | 1 |  |  | 1 |  |  |  |
| Soft Stool | 1 | 1 |  |  |  |  |  |
| Dysuria | 2 | 2 |  |  |  |  |  |
| Pollakisuria | 2 | 2 |  |  |  |  |  |
| Infection (e.g. bladder) | 3 | 3 |  |  |  |  |  |
| Fever | 2 | 2 |  |  |  |  |  |
| Oral mucositis | 2 |  | 2 |  |  |  |  |
| Nasal mucositis | 2 | 2 |  |  |  |  |  |
| Weight loss | 1 | 1 |  |  |  |  |  |
| Odynophagia | 1 |  | 1 |  |  |  |  |
| Fagitue | 7 |  | 7 |  |  |  |  |
| Paresthesia/Hypästhesia | 2 | 2 |  |  |  |  |  |
| Joint range of motion decreased | 2 |  | 2 |  |  |  |  |
| Conjuctivitis | 1 | 1 |  |  |  |  |  |
| Gastrointestinal bleeding |  |  |  |  |  |  | 1 |
| Decreased visual acuity | 1 | 1 |  |  |  |  |  |
| Pain | 12 | 11 | 1 |  |  |  |  |
| Stridor | 1 | 1 |  |  |  |  |  |

| **Supplemented Table 2:**  **Late toxicity (> 3months)** | | | | | | |
| --- | --- | --- | --- | --- | --- | --- |
| Toxicity | All | Present | CTCAE Grade | | | |
|  |  |  | I | II | III | |
| Fatigue | 7 | 3 | 4 | 1 |  | |
| Fibrosis | 4 | 2 | 1 |  | 1 | |
| Lymphedema | 8 | 7 | 1 |  |  | |
| Pain | 18 | 11 | 5 | 1 | 1 | |
| Hyperpigmentation | 10 | 7 | 3 |  |  | |
| Induration of the subcutaneous tissue | 3 | 3 |  |  |  | |
| Teleangiectasia |  |  |  |  |  | |
| Arthrosis with joint replacement | 1 | 1 |  |  |  | |
| Joint range of motion decreased | 7 | 6 | 1 |  | |  |
| Joint effusion | 1 | 1 |  |  | |  |
| Neuropatic pain | 2 | 2 |  |  | |  |
| Spasticity | 1 | 1 |  |  | |  |
| Radiodermatitis | 1 |  | 1 |  | |  |
| Sensory disturbance | 2 | 2 |  |  | |  |
| Paresis | 2 | 2 |  |  | |  |
| Sepsis | 1 | 1 |  |  | |  |
| GI fistula | 3 | 3 |  |  | |  |
| GI perforation | 1 | 1 |  |  | |  |
| Chylous aszites | 1 | 1 |  |  | |  |
| Portal vein thrombosis | 1 | 1 |  |  | |  |
| GI abscess | 2 | 2 |  |  | |  |
| GI bleeding | 1 |  | 1 |  | |  |
| Rectal ulcer | 2 | 2 |  |  | |  |
| Marisca/ anal fisur | 1 | 1 |  |  | |  |
| Soft stool | 1 | 1 |  |  | |  |
| Climacterium | 1 | 1 |  |  | |  |
| Reduced physical capacity | 1 | 1 |  |  | |  |
| Anosmia | 1 |  |  | 1 | |  |
| Dysgeusia | 1 | 1 |  |  | |  |
| Congitive impairment | 1 | 1 |  |  | |  |
| Dry nasal mucosa | 1 | 1 |  |  | |  |
| Dysphagia | 1 | 1 |  |  | |  |
| Trismus | 1 | 1 |  |  | |  |
| Seroma | 1 | 1 |  |  | |  |

| **Supplemented Table 3:**  **Univariate analysis for PFS,** unifocal localization, no previous medical treatment, dose above > 50 Gy, and age >41y were associated with improved survival | |
| --- | --- |
|  | **p-value** |
| Gender | 0.641 |
| FAP | 0.158 |
| Unifocal vs. multifocal localisation | 0.013* |
| First diagnosis vs. recurrence or progression | 0.494 |
| Definitive vs. postoperative radiotherapy | 0,306 |
| Macroscopic tumor on planning computed tomography vs. no macroscopic tumor | 0.481 |
| Applied dose $\leq$ 54 Gy/Gy(RBE) vs. > 54 Gy/Gy(RBE) | 0.068 |
| Applied dose $\leq$50 Gy/Gy(RBE) vs. > 50 Gy/Gy(RBE) | 0.014* |
| Planning target volume (PTV) $\leq$967 ml vs. >967 ml | 0.376 |
| Localisation abdominal vs. extraabdominal | 0.511 |
| Previous medical treatment, yes vs. no | 0.035* |
| Previous surgery vs. no pervious resection | 0.444 |
| Age, | 0.002* |
| Particle therapy vs. photon radiotherapy | 0.588 |
